# Family food practices: relationships, materiality and the everyday at the end of life

**DOI:** 10.1111/1467-9566.12606

**Published:** 2018-02-21

**Authors:** Julie Ellis

**Affiliations:** ^1^ Department of Sociological Studies University of Sheffield UK

**Keywords:** Food, family practices, relationality, end of life, everyday life, materiality

## Abstract

This article draws on data from a research project that combined participant observation with in‐depth interviews to explore family relationships and experiences of everyday life during life‐threatening illness. In it I suggest that death has often been theorised in ways that make its ‘mundane’ practices less discernible. As a means to foreground the everyday, and to demonstrate its importance to the study of dying, this article explores the (re)negotiation of food and eating in families facing the end of life. Three themes that emerged from the study's broader focus on family life are discussed: ‘food talk’ and making sense of illness; food, family and identity; and food ‘fights’. Together the findings illustrate the material, social and symbolic ways in which food acts relationally in the context of dying, extending conceptual work on materiality in death studies in novel directions. The article also contributes new empirical insights to a limited sociological literature on food, families and terminal illness, building on work that theorises the entanglements of materiality, food, bodies and care. The article concludes by highlighting the analytical value of everyday materialities such as food practices for future research on dying as a relational experience.

In death studies there are a number of empirical accounts of dying as a social, embodied process (Hockey 1990, Lawton 2000), whilst more recently sociological literature concerned with family and relationality at the end of life has started to emerge (Broom and Kirby 2013, Ellis 2013, Woodthorpe and Rumble 2016). Although this, and some previous work, examines ‘family’ in the context of life‐limiting/threatening illness (Bluebond‐Langner 1996, Grinyer 2002), the conceptual relevance of everyday practices for understanding how relationships are negotiated when a relative is dying has received limited attention. As I have argued elsewhere (Ellis 2013), notions of crisis and rupture influence theorising in death studies and contribute to a discursive framing of the end of life that often foregrounds the extraordinary rather than the more mundane and everyday. As Kellehear (2014: x) points out:

Although dying is commonly portrayed as a dramatic affair, a problematic thing, a medical experience, it commonly plays out against type. More often than many like to think, dying is a surprisingly quiet affair … Most books on dying often simply fail to provide a description of this handful of usual experiences.

To explore how the ‘ordinary’ can illuminate experiences of dying, this article analyses data about food that emerged from a research project on everyday family life during life‐threatening illness. The findings suggest that food practices play a part in the ‘doing’ of family (Morgan 1996, 2013) at end of life that is simultaneously material, symbolic and social (Abbots and Lavis 2013). This is identified in the following three themes: ‘food talk’ and making sense of illness, food, family and identity, and food ‘fights’.

Whilst previous work on death has explored material culture mainly in connection to relationships with the deceased, the article extends this focus on materiality in new directions, examining how food as an aspect of material culture shapes relationships before death, over the dying process. It also makes an empirical contribution to a limited sociological literature on food, families and terminal illness, building on recent conceptual work that theorises the entanglements of materiality, food, bodies and care. By examining food and families near the end of life, the article highlights the more general importance of everyday materialities for understanding dying as a relational experience.

## Relationships, materiality and the end of life

Family life is characterised by the habitual. It is where the everyday and its material components traverse in repertories of routine. As Morgan (2004: 40) explains, ‘[f]amily practices are organised around the regular deployment of bodies, time and space and material culture’. The importance of ‘matter’ in the constitution of relational life is well documented. Miller (2008: 286) argues that objects are ‘an integral and inseparable aspect of all relationships’, whilst Smart (2007) stresses the materiality of relational feelings in kinship. Material things are also significant in carer‐patient relationships as van Hout *et al*. (2015) describe in their study of home care visits. Drawing on participant observation and interviews with oncology nurses and patients they identify ‘how materialities and people shape home care together’ (van Hout *et al*. 2015: 1209). In attributing agency to non‐human entities this analysis resonates with a material turn across the social sciences that recognises matter as ‘processual, emergent, and always relational’ (Lavis and Eli 2016).

There is a body of literature on the material culture of death which attests to the relevance of these observations for post‐mortem relations between the living and deceased. A number of social and anthropological studies of bereavement describe how relationships are embedded within and affected by material, spatial and sensory dimensions of everyday life (Hockey *et al*. 2010). Richardson's (2014: 72) study focuses on the home in particular as a site of memory where ‘social and sensory connections can be made with a deceased spouse through an embodied relationship with materialities such as clothing, photographs and furniture’. In interviews bereaved spouses also discussed their (re)enactment of mundane practices (hanging curtains, folding bedsheets) intimating that a sense of connection to the deceased is experienced *corporeally* by survivors during the performance of these everyday tasks. Miller and Parrott (2007) similarly describe how ‘ordinary’ materialities offer opportunities for ‘domesticating’ and personalising bereavement. They argue that items of domestic material culture (CDs, clothing, photographs) enable bereaved individuals to ‘fulfil specific commemorative needs’ that are important for the construction of personal rituals which represent the individuality of the deceased and the mourner's relationship with them (Miller and Parrott 2007: 159).

Therefore objects have the ability to embody traces of the dead whether materially (e.g. clothes worn against bodies) or via associations and memories (Gibson 2008). Whilst bereaved individuals can use ‘stuff’ to actively construct ongoing relationships with the deceased, this agentic potential can flow both ways with ‘things’ precipitating unanticipated moments of ‘extreme remembering’ (Heldke 2016). In a personal account about the loss of her parents Heldke (2016: 88) explains how ‘my moments of sharpest grief, as well as my moments of most vivid connection with them, were always occasioned by food’. She describes visceral experiences of encountering certain foods and the vivid memories they invoke via sight, smell, taste and touch. Here Heldke provides a clear example of memory *as* sense (Korsmeyer and Sutton 2011) – ‘a creative channel between present and past that enables the past to both suffuse the present and inflect the future’ (Heldke 2016: 88). In this way, food as a material trigger for memory is a less obvious example of what Hallam and Hockey (2001: 51) have conceptualised as ‘emergent memory objects’. That is, things that have connections to life histories and which ‘are able to condense different times through their aesthetic, sensual or material properties’.

Notwithstanding this insightful work on memory, materiality and bereavement, conceptual and empirical accounts of materialities and social relationships over the dying process are more limited. Whilst studies have explored the physical environment in which palliative care takes place (McGann 2013, Tishelman *et al*. 2016) and there is some research on personal objects in care settings (Kellehear *et al*. 2009), how family relationships towards the end of life are mediated by materiality requires sociological exploration. In this article I seek to do this by focusing on food practices.

## Families, food and terminal illness

Eating is an important family practice (Morgan 1996, 2013). It is primarily within families where food preferences, eating‐related behaviours and identities are acquired and experienced (Lupton 1996, Valentine 1999). It is also where we learn to enjoy (although not always) the bodily experience of food – how it tastes, smells and feels – its material, sensory and affective aspects (Abbots and Lavis 2013). In this respect, food has distinctive properties as material culture – it is less solid or stable than many material things, and can be incorporated directly into corporeal bodies and their organic systems (Bennett 2007; Roe 2006). Because food can be processed in a multitude of ways (e.g. cooked, canned or chopped), it exemplifies ‘becoming over being’ (Bennett 2007:135). It is a pertinent example of how materialities (including bodies) ‘become together’ – in an agentic, processual sense (Lavis and Eli 2016). Roe (2006: 467) considers the emergent materiality of food in a study of how genetically modified foods become inedible, and in the context of serious illness it is useful to consider her assertion that ‘the quality of the material is significant to the construction of edibility’. For individuals experiencing the embodied flux of disease, certain foods – their textures, tastes, smells, appearance – acquire altered ‘material signifiers’ that challenge and reconstruct their ‘edibility’ (Roe 2006: 473). Therefore conceptual approaches that interrogate the entangled materiality of food/bodies can offer insight into the embodied experience of terminal illness.

Recently, food scholars have theorised the materialities of care and eating, pointing out how acts of caring for another's body – such as feeding – can involve (and therefore ‘make’) multiple bodies (Lavis *et al*. 2015: 10). They also suggest that eating and caring can blur corporeal boundaries creating relational possibilities for both intimacy and tension between feeders and eaters. In the context of families dealing with terminal illness where the morality of care is writ large (Broom *et al*. 2016), these issues are thrown into particularly sharp focus but receive limited attention in the sociological food literature. Lavis *et al*. (2015: 4) do not recognise dying bodies explicitly when they argue that ‘in individual moments of eating and feeding, care may not always be felt to be so ‘caring’, or food so ‘good’ or ‘right’’. However, what they argue regarding the ‘slipperiness’ of care's assumed banality can illuminate findings cited in other literatures where food is identified as a source of conflict for families experiencing terminal illness (Hughes and Neal 2000).

As one of the few sociologists to write about food and dying specifically, Seale (1998: 160) recognises that, ‘[a]s the material precondition of existence the gift of food is readily equated with the gift of life’. Therefore in this context feeding and eating involves making and remaking social relations between self and other (Lavis *et al*. 2015). In her sociological analysis of food and fluids in the care of terminally ill patients, McInerney (1992) identifies these processes working at a cultural, discursive level. In this setting she argues that the symbolic nature of food is suffused with that of ‘family’ and these operate powerfully together to make feeding an emotive issue and a clinical enactment of nurturing. Thus, according to McInerney, pervasive ideas about compassion and care intrinsic to the symbolism of food and families underpin clinical decisions to administer artificial nutrition in circumstances deemed to be biologically futile. Harbers *et al*. (2002), discuss similar issues regarding feeding in a Dutch nursing home. Their focus is on an ethnographic exploration of the ‘socio‐materiality’ of food – revealing relational aspects of daily care that complicate the impasse of ethical and biomedical versions of what is ‘right’ or ‘good’ in these circumstances. Crucially these, and related issues regarding nutritional care in hospitals for frail, older people (Heaven *et al*. 2013) continue to be deliberated with much emotion as the recent controversy in England surrounding the Liverpool Care Pathway (LCP) demonstrates.^1^

Hence there is a need for more sociological attention to be paid to food, eating and the materiality of both, in end of life contexts. Particularly as the salience of food for families living with terminal illness has emerged recently as an area of research in palliative and allied literatures (see Raijmakers *et al*. 2013, Reid *et al*. 2009, Wallin *et al*. 2013, 2015). This developing body of work identifies a number of issues which individuals and their families might face. These include: anorexia, malnutrition, reduced appetite, weight‐loss, disruption to shared food practices, interpersonal conflict and implications for sense of self and identity. Sociological studies of individuals with less advanced, life‐threatening cancer (Mróz *et al*. 2011, Wainwright *et al*. 2007) and chronic illnesses such as coronary heart disease and coeliac disease (Gregory 2005), also provide important broader insights into how illness and its treatments can affect eating as a relational experience. Nonetheless, there is little sociological literature on food, families and terminal illness specifically – which is the focus of this article.

## The study

This article is based on a subset of findings from a project about everyday family life during life‐threatening illness (Ellis 2010). An objective of the research was to study family experiences in an exploratory, non‐retrospective way over the illness/dying process. Appropriate National Health Service (NHS) ethical approvals were sought to recruit participants via a hospice in the north of England and repeat, in‐depth interviews were conducted with families attending the hospice's day care service. This generated data about family life when the ill person's disease was less advanced, whilst participation observation on the hospice's inpatient unit was undertaken to observe families closer to death.

Patients attending the day care service were approached by a lead nurse and offered information about the research. Those interested in taking part were asked to invite relatives they saw regularly to join the study. Nine families (nine patients, fourteen relatives) participated in a combined total of 39 interviews. The benchmark of three interviews over a few months was initially suggested, however, the number completed with each family differed depending on circumstances (including speed of disease progression).^2^ Participants were asked if they wanted to be interviewed alone or with other family members. All those living together requested to be interviewed together and most interviews took place in family homes. At initial interviews informed consent was gained and a topic guide used to ask questions about the family's background, present day‐to‐day life (e.g. cooking, housework, holidays) and effects of the illness. The purpose of any subsequent interviews was to talk about changes, and to follow up salient issues discussed on previous occasions.

In addition, I completed 175 hours of participant observation on the hospice's inpatient unit, taking handwritten field notes and then fleshing these out more fully after visits. On the ward I performed the duties of a volunteer, chatting to patients, relatives and staff. The unit had facilities to care for up to eight patients, with four private rooms and a shared four‐bedded area. The majority of patients had terminal cancer, with some receiving care at the very end of life and others admitted for symptom monitoring. Due to the constant comings and goings of patients and visitors, it was impractical to gain written informed consent. Instead, information sheets were disseminated by volunteers on reception as visitors arrived at the hospice, and I offered these to patients. I then had follow‐up conversations to discuss consent. In cases where patients lacked capacity this required sensitive negotiation with families. If relatives stated they wanted to be involved, they became my main focus in observations and consent was continually revisited.

To analyse the data I used Nvivo (QSR International, Brisbane) and the coding process was informed by a thematic approach to narrative analysis, where a participant‐centred focus was retained, despite looking for themes across the entire dataset (Riessman 2008).

## Findings and discussion

### ‘Food talk’ and making sense of illness

Observing life on the hospice inpatient unit it became apparent that food had a prominent material and symbolic presence. In hospitals and similar institutions food is typically managed in back stage areas, although this was not the case on the hospice ward. Here meals were presented to patients on trays and food was often served in a communal area. At mealtimes a large, wooden dresser (side‐board) acted as a material centrepiece around which the orchestration of feeding unfolded, with staff and volunteers taking items of crockery and cutlery from the dresser to make‐up trays for patients in a manner not unlike laying the table at home. Food was therefore awaited, prepared for and served, publically. All this activity was a visible marker of routine which not only gave a sense of structure to the day – these care practices also involved the ‘practical arrangement’ of various materialities (van Hout *et al*. 2015) that imbued the provision of food with an embodied intimacy.

On the inpatient unit, ‘food talk’ also provided a focus around which interactions between patients, their families and staff members took place. What their ill relative had managed to eat was something families often wanted to know; eating was viewed positively and welcomed with a sense of relief (Wallin *et al*. 2013, 2015). Some family members also tried to tempt patients to eat, reflecting what Seale (1998: 164) describes as ‘temptations to life’. An exchange with Mabel, a woman in her 60s who came to visit her husband, reflected this.

Mabel was overjoyed about … the fact that he'd had a FULL (emphasis placed on this) breakfast. She tells me that he hasn't been eating a thing and how worrying this has been; she has been making him jellies and buying ice cream – anything to try and ‘tempt’ him, but he hasn't been interested (field notes).

Similarly, during in an interview with Helen (69 years) and her sister Vera (76 years) who had lung cancer, Helen spoke about how she tried to ‘tempt’ Vera's fluctuating appetite.

Helen Well when she was diagnosed at first she had no appetite at all … her appetite was terrible, it was, I was at my wits end I was trying to think up things that would tempt her to eat and things that she would maybe like – a little bit of fish and toast or – and then they put her on these steroids that's wonderful she'll just eat anything and that's great, absolutely great.

And so, when an ill relative struggled to eat, families felt particularly concerned. An exchange between Mabel (introduced above), and her husband Rob conveys this.

I sense tension between them as Mabel tries to ask what Rob's eaten today. We tell her that he's eaten some meat and potato pie – Rob adds that he didn't have any meat though because it's too rich … She asks if he ate all the pie and Rob says not. She also asks about pudding and learns that he hasn't had any. There is a bit of a silence and she concludes that he hasn't had much again then – making a dry comment about him aiming for the catwalk and then clarifying what she meant by explaining he was trying to get to a size zero. Beneath the banter and the brave face it is easy to see how worried Mabel is about Rob (field notes).

Here it is apparent that Mabel is trying to gauge what Rob has eaten in her absence and that eating is an ongoing worry – something Mabel is actively trying to monitor. She uses euphemisms to insinuate symbolically her fears about Rob's wasting body; refraining from stating directly what this might infer about his disease progressing. For Rob his focus is more with the materiality of the task of eating, describing how the distinctive properties of particular foods preclude their ability to be eaten by him. The meat in the pie is untouched because it is too ‘rich’; a decision most likely shaped by Rob's past ‘visceral and corporeal knowledges’ of meat (Roe 2006: 477) which inform what he feels he can eat now.

Because certain foods become associated with people and their habitual practices, these could also offer a way to think about and make sense of illness for families. The following notes describe a conversation with a couple in their 70s who were discussing John getting ready to go home after a stay in the hospice.

His wife tells John that she thinks he will be alright now – now he has seen some improvement. I was struck when she said that his bottle of whiskey will last him beyond Christmas – when a few weeks ago she didn't think he would make it until then … John didn't comment (field notes).

For John's wife this whiskey symbolises a sense of who he is (‘his bottle of whiskey’), and its physicality (what is in the bottle) is a rather ‘ordinary’ but meaningful way for her to make sense of the illness and its progression. In other words, its materiality becomes a means to think about a life with John beyond Christmas – the liquid in the bottle representing ‘measures’ of life.

Collectively what these examples begin to show is how food – symbolically and materially – becomes a way of thinking about, monitoring and making sense of illness, representing an interlacing of the day‐to‐day with the relational negotiation of life‐threatening illness.

### Food, family and identity

There is a substantive body of research that attends to how everyday relationships with food shape individual and collective social identities (Caplan 1997, Fischler 1988, Nettleton and Uprichard 2011). Experiencing serious illness can incline a corporeal state that is quite literally unable to stomach eating norms, and as such, can effect an individual's sense of self and how others relate to them (Reid *et al*. 2009, Wainwright *et al*. 2007).

For Eddie (78 years) living with stomach cancer meant food was particularly problematic. When I first met him and his wife Kathleen (75 years) I was shown a photograph and asked to note the physical changes a lack of appetite had made to Eddie's appearance. Eating then became a focus in the interview, as the couple discuss below.

Julie And did you used to like your food Eddie?

Eddie Oh aye I was always a big eater

Kathleen Yeah! He used to have platefuls – he loved his dinner – he won't touch Yorkshire pudding anything like that [now] – bacon and egg

Eddie I'd have two or three dinners me (pause) but not now it takes me all my time trying to get one down now.

Here Kathleen refers to certain foods which seem symbolic of Eddie's dwindling appetite and dissipating identity as a ‘big eater’. She mentions ‘hearty’ foods with regional and gendered connotations that evoke a specific connection to an eater that is lost, or at least slipping away. In this sense it becomes possible to conceive of these foods as everyday ‘emergent memory objects’ (Hallam and Hockey 2001) which have agency to prefigure anticipation of more embodied extremes (Lavis and Eli 2016) that might await in the imminent future.

Indeed, the idea that Eddie was no longer a ‘big eater’ was significant for this family's experience of the end of life, as it was also mentioned by his children. The couple's daughter Laura (49 years), used the term ‘odd’ to denote her awareness of his embodied transition.

Laura Yeah cos at Christmas to be honest I mean I didn't think he'd be here at Christmas … he sat and had his dinner and he only had a right little bit but I mean like now its soup … then he were being able to eat little bits of things … it's odd because when you have … always known him to be a big eater and like now … he's so thin now.

The shifts in how Eddie is known within his family as a particular kind of eater are signified by the changing material status of food he is able to consume, as well as his physical appearance. Laura notes how as time passes and Eddie's disease progresses, the food he eats is less meal‐like in composition and less stable in material form. Thus as his food becomes more fluid, so does his physical and social identity.

At this time Kathleen's identity as a particular ‘kind’ of food provider was also in flux; something that happened in both material and symbolic terms. As Laura explains she and Kathleen were involved in conversations about the appropriateness of certain foods and preparation techniques. They debated what might be the most suitable material properties (textures, tastes) for Eddie's fluctuating sense of edibility (Roe 2006).

Laura Well all he can have is soup and me mother kept ringing me up and [saying] ‘he's not eating, he'll not have anything I make him, he's not eating it’ and so I says to her ‘why don't you make him some soup?’ And me mum's one of these traditional people … she's old fashioned that she thinks everything should be fried. And I said to her ‘why didn't you do him a liver casserole and do it in the oven?’ and she didn't seem to get her head round it …

Here Laura provides an explanation of how she understood what was needed to produce something that would become more edible for Eddie (Roe 2006). This involved working with the materiality of different foods and methods of production (blending, baking or frying). In other words, processes of tinkering and adapting materiality (van Hout *et al*. 2015). At a more symbolic level her ‘tinkering’ undermined Kathleen's familial food knowledge (Morgan 1996), exposing her practices as ‘old fashioned’. The significance of this for Kathleen as a food provider was discussed by the couple's younger daughter Claudia (37 years) and her daughter Joanna (13 years).

Claudia But it's not very often that she does that now is it cos she's not making it (dinners) … but whereas me mum religiously it didn't matter if it were 100 degrees outside there was always a cooked dinner on the table whereas with now

Joanna [A] proper cooked dinner … Sunday dinner, proper Yorkshire puddings, beef, veg – proper

Claudia It were like meat, potatoes and veg that's me mum, that's what me mum's always done – do you know what I mean? … It's me mum's era … 50s housewife weren't it? How to look after your husband.

What they describe here reflects cultural ideas about how particular foods and their combinations constitute a ‘proper’ meal (Douglas 1975) and how these configurations become embedded in the production (symbolic and material) of family life (Charles and Kerr 1988, DeVault 1991). For Kathleen, providing a ‘proper’ meal for her family seemed important, or at least it was for how others identified and related to her as a feeder.

In a different family, Dot's (76 years) husband Hugh (69 years) was living with lung cancer and she was also adjusting to his preference for ‘lighter’ foods rather than meals. For Dot feeding was an act of care, and she became frustrated when food available at the day hospice Hugh attended seemed to more ‘care‐fully’ meet his needs (Lavis *et al*. 2015:7).

Hugh Well this is it she's always onto me. I mean she's had a go at me today – ‘you are eating that food up at the hospice you won't eat what I'm cooking’ …

Julie Does it make you feel a bit because you like to care for Hugh … does it make you feel a little bit

Dot I like to cook me own you know for em (him) and I think well he's going out and he's eating other people's – I know he's got to – and he knows they'll make him

Hugh They won't make me love. I have what I want up there (at the hospice). If I don't want it up there I can have a sandwich … if she brings the menu I say ‘no there's nowt I fancy there love and I'm not hungry for owt like that’ I could have a sandwich or owt you know…

Dot Oh that's why you keep asking me for sandwiches then when you are not

Hugh No

Dot Cos you never used to did you? You always had a big meal

As Dot explained, she liked to ‘cook me own’ – a phase which connotes clearly the embodiment of ‘self’ in family food practices. Moreover, what this exchange also reveals is how the ambiguities both faced as a result of the illness were manifested materially in the contentious qualities of a sandwich. For Hugh this lighter snack embodies the flexibility his fluctuating appetite demands. It is also versatile and quick and easy to prepare – properties which, it would seem, make for a less pressured feeding and eating relationship. For Dot the sandwich's materiality means the inversion of this; it embodies an agency that challenges what she knows about Hugh as a particular kind of eater and herself as feeder, destabilising predictable ‘socio‐material relationalities of eating and caring’ (Lavis *et al*. 2015:3).

The data presented in this section have shown, therefore, that the material entanglements of food, bodies and care in the context of day‐to‐day experiences of terminal illness, mediate social processes of identification (Jenkins 1996). What these findings also bring to the fore is the everyday contexts in which these relational experiences of identity are embedded and negotiated.

### Food ‘fights’: conflict and tension

When Claudia told me that for her father Eddie, food became ‘the root of evil’, she echoed existing research findings that have identified food as a cause of conflict in families living with life‐threatening illness (Hughes and Neal 2000). Specifically my research revealed eating as an embodied, relational practice that could span across several households, shaping the conversations, concerns and actions of different family members. Not only did this reveal the contentious ‘matter’ of food, it also demonstrated how eating is a shared, ‘multiple’ practice involving various different people (Lavis *et al*. 2015: 10). For example, in an interview with Cindy (36 years) I learnt about some of the food‐related ‘tussles’ involving her older sister Sue (45 years) and their mother Anna, (68 years) who had a chronic auto‐immune condition as well as breast cancer.

Cindy cos me mum keeps saying ‘our Sue won't leave me alone, she won't leave me alone, she doesn't realise what I eat’ … you get to learn what me mum can eat. I mean she's been through all these dieticians and things, but I do feel sorry for our Sue cos she (mum) is right hard and she is right trying … and I think yeah I agree with our Sue she does need to eat more but our Sue needs to think hang on a minute she'll not be able to digest that … Alright she's not getting all the vitamins she needs but I just think while she's poorly let her eat what she wants – a bacon butty with tomato on it or something you know whatever but our Sue's trying to shove all these noodles and things down her (laughing)

What Cindy describes here echoes Laura's earlier account of knowing best how to ‘tinker’ with Eddie's food so it would become more edible. Cindy distinguishes between particular foods she thinks Anna finds appealing/edible but which are deemed less nutritious, and more ‘healthful’ items such as noodles preferred by her sister. Thus the material properties of foods embody the sisters’ different ideas about what is the ‘best’ and most ‘care‐full’ approach to Anna's eating (Lavis *et al*. 2015) – whether to prioritise nutrition or sensorial things like ‘comfort’ or taste. Here Cindy is able to subtly infer her own ideas as being more attuned than her sister's when it comes to understanding Anna's changing bodily needs (note the reference she makes to digestion), signifying how differential positions of power meet and jostle at the material intersections of ill bodies and food.

In Eddie's home the sensory aspects of food preparation became a visceral challenge when he complained that the smell of cooking made him feel sick. Therefore Claudia described how at times Kathleen would avoid cooking and shopping for ‘proper’ food, as she was generally less motivated to make meals just for herself. This made Claudia and her siblings (including an older brother) worry about the potential impact on Kathleen's own health. With Claudia living only minutes away from her parents and calling to see them most days, she consequently felt under pressure to make Kathleen an evening meal.

Claudia And I mean it's like our Laura says to me the … other week … ‘why don't you start cooking for me mum?’ And I went ‘Laura I haven't got a problem cooking for me mum … ‘but’ I says – me mum's like religiously dinner's been on the table like between 1 and 2, I says ‘I don't eat like that Laura’ I says ‘I work just like you work’ … it can be sometimes 8 o'clock for me to cook a proper dinner it's time consuming do you know what I mean? … I says ‘I have even offered to plate it her up but she won't have it warmed up’.

Here the latent agency of everyday ‘matter’ in the arrangement of family practices is made apparent in Claudia's frustration that Laura fails to consider that cooking a ‘proper dinner’ for Kathleen would entail re‐organising the temporality of eating within her own home to accommodate her mother's ideas about when and how meals should be eaten. In this, and a further example where Claudia expresses her irritation that Eddie could (but does not) go upstairs to avoid cooking smells, the family's situation illustrates how tensions are embedded within spatial and temporal aspects of daily family life and the material and sensory properties of food which permeate these.

Finally, as an earlier example has already suggested, for Hugh his dwindling appetite caused friction between himself and his wife Dot as it interrupted habitual eating patterns they had established over years together.

Dot Well yeah you've not done too bad – yeah but when you've had toast you don't have nowt no more and unless I decide to cook

Hugh I know it's getting you to cook now and again (joking)

Dot No it isn't it's getting you to eat it, int it? I'm going to start and get some tins of soup in …

Hugh I don't want soup; I'm not a soup fan I never have been!

Dot I know you're not

Hugh Well I don't want soup

Dot I mean I have got a freezer full of meat in there, joints of meat I've chucked, today I have thrown half of one away I cooked other day…Big piece of beef; I cook it and it just gets thrown away. On a Sunday I always cook one on a Sunday, he'll have one piece I have about the same and the other goes in the bin. Dustbin gets more than us.

There are hints of ‘tetchiness’ in this exchange when Hugh protests at the idea that Dot might serve him soup and Dot signals her frustration by ‘threatening’ to stock up on the very item Hugh says he does not want. Soup is materially ambiguous as food given its liquid form – something which in the context of this conversation seems to symbolically represent the resignation Dot feels about her role as a food provider. This was conveyed more clearly at another point in the interview when she said there was no point in doing ‘proper’ cooking now. On one level the couple are unhappy about the amount of food they are wasting, which is made apparent to them in a material sense when quantities of food are thrown away. Symbolically, what this waste (the full dustbin) also represents, however, is a shift in how they once knew one another via their predictable eating patterns. Thus in their day‐to‐day interactions around food and the friction this sometimes created, frustrations seemed to stem, in part at least, from having previously experienced eating – that is ‘proper’ meals – as constitutive of family life (Charles and Kerr 1988, DeVault 1991).

What these dynamics of tension begin to show is the ‘slipperiness’ of care in relation to food (Lavis *et al*. 2015). In other words, these accounts complicate idealised notions of ‘family’ and ‘care’ which are made all the more normative in the context of end of life (Broom *et al*. 2016) but which have not been explored so explicitly in sociological research on food. What this last theme starts to map out, therefore, is how ‘food fights’ reflect attempts to care for others through the embodied acts of feeding and eating. Practices which in the unfolding of day‐to‐day at the end of life are not always ‘experienced as caring or care‐full’ in any given or straightforward way (Lavis *et al*. 2015: 7). The findings also build on and extend existing research, by teasing out the mundane ways in which these slippery dynamics play out between multiple individuals/ bodies within a family, rather than unilaterally between a primary ‘carer’ and ill person.

## Conclusion

As a way to foreground the everyday, and to demonstrate its importance to the study of dying, this article has examined materialities of food and eating, highlighting their social and symbolic significance for families living with terminal illness. Specifically the findings have shown how food enables people to think about, monitor and make sense of the embodied illness of a family member, representing one way in which ‘mundane’ materialities play a meaningful part in negotiation of dying as an everyday experience (Ellis 2013). A further way in which the data demonstrates this is in instances where the materiality of food, bodies and care become entangled and reveal eating‐related social identities to be in flux and requiring negotiation. The uncertainties these processes create for family life have also been explored using examples in the data that demonstrate food can become a contested and conflictual everyday ‘matter’ for families.

Focusing on family relationships at the end of life the article has therefore elucidated how eating becomes an act that is both ‘multiple’ and ‘slippery’ (Lavis *et al*. 2015) in a context that has not been adequately explored in the sociology of food literature. Collectively the findings demonstrate food's ‘agentic capacity’ to affect relational experiences of life‐threatening illness – providing an original empirical illustration of Bennett's (2007: 134) assertion that food is ‘a co‐participant in our world’. By building on recent work in food studies that theorises materiality, food, bodies and care, this article extends the conceptual analysis of materiality in death studies beyond bereavement. By focusing explicitly on food and its constitutive role in relationships over the dying process it points to new directions for further sociological inquiry. Having identified food as a particular material focus and demonstrated the material, social and symbolic ways in which it acts relationally in the context of dying, I argue that this article highlights the more general importance of everyday material practices for future research on the end of life. The analysis developed here suggests there is merit in paying analytical attention to other everyday materialities – objects, spaces and temporalities of daily life – to further develop sociological scholarship on dying as a relational experience.

## Notes

1 The LCP was a tool designed to facilitate high quality end of life care. It came under scrutiny in 2013 when an independent review ruled it should be abolished.

2 I conducted post‐death interviews with two families.
